# A Detection and Tracking Algorithm for Resolvable Group with Structural and Formation Changes Using the Gibbs-GLMB Filter

**DOI:** 10.3390/s20123384

**Published:** 2020-06-15

**Authors:** Xinfeng Ru, Yudong Chi, Weifeng Liu

**Affiliations:** College of Automation, Hangzhou Dianzi University, Hangzhou 310018, China; a840064210@hdu.edu.cn (X.R.); cyd0418@163.com (Y.C.)

**Keywords:** target tracking, group targets, GLMB, structure, formation

## Abstract

In the field of resolvable group target tracking, further study on the structure and formation of group targets is helpful to reduce the tracking error of group bluetargets. In this paper, we propose an algorithm to detect whether the structure or formation state of group targets changes. In this paper, a Gibbs Generalized Labeled Multi-Bernoulli (GLMB) filter is used to obtain the estimation of discernible group target bluestates. After obtaining the state estimation of the group target, we extract relevant information based on the estimation data to judge whether the structure or formation state has changed. Finally, several experiments are carried out to verify the algorithm.

## 1. Introduction

In many fields, the application of target tracking technology can be seen. For example, target tracking is an important part in the field of self-driving cars. Self-driving cars technology is one of the research focuses at present. In the process of driving a car, its control system needs to detect and discriminate the surrounding environment of the vehicle, and target tracking technology plays an important role in this part [1]. Target tracking technology can also be used in the field of national defense such as aircraft tracking in the air, ships tracking at sea, and vehicles tracking on land. Usually, they coordinate their movements in a certain way. Scholars have made many excellent achievements in the field of target tracking, among which the research on multi-target tracking based on random finite set (RFS) is also very fruitful; such as machine learning [2], computer vision [3], autonomous vehicle [4], sensor scheduling [5,6,7,8,9,10,11,12], sensor network [13,14,15], blue, in particular, a fast RFS based distributed tracking algorithm is presented for a sensor network in [15] and track-before-detect, tracking of merged measurements, and target tracking [16,17,18,19,20].

Mahler is the first one to apply the RFS theory to the field of target tracking [21,22,23,24] and gives the probability hypothesis density(PHD) filter [22,25], the cardinalized PHD (CPHD) filter [26,27], and the multi-target multi-Bernoulli (MeMBer) [28]. The MeMBer filter is different from the PHD filter and the CPHD filter. The MeMBer filter propagates the parameters of a multi-Bernoulli distribution that approximate the posterior multi-target density and the others propagate the moments and cardinality distributions. Subsequently, the CBMeMBer filter [29] is proposed to solve the problem of cardinal deviation of MeMber filter. References [30,31] introduce label into RFS and propose a Generalized Labeled Multi-Bernoulli (GLMB) filter. Reference [30] gives the implementation method δ-Generalized Labeled Multi-Bernoulli (δ-GLMB) filter of GLMB, and the truncation method is used to refine the δ-GLMB in Reference [31]. In Reference [32,33], Vo et al. simplified the prediction step and update step of GLMB filter into a single step. This new method to implementation is known as Gibbs GLMB. The Gibbs-GLMB filter greatly reduces the computational complexity of GLMB filter and improves the computational efficiency. Based on these filters, some people have done some work on tracking hybrid targets. Hybrid targets is a collection of multiple target, group target and extended target. The problem of tracking multi-measurement targets is concerned in [34]. In Reference [35], group target tracking in the case of uncertain number of targets and groups. Reference [19] focuses on dynamic modeling and tracking estimation for multiple resolvable group targets. Reference [36] considers group state estimation. Under the framework of RFS, the work of modeling and tracking estimation for multi-extended targets is contributed in the Reference [37]. In the Reference [33], we apply a Gibbs-GLMB filter to estimate the state of resolvable group targets and track them.

In this paper, based on the collaborative relationships between targets and the state information of each independent target, we propose a method to determine the structure and formation change of resolvable group targets. We call the dependency relationship between group targets as structure and the shape formed by group targets with fixed spatial distance as formation.

The content of this paper is arranged as follows: Section 2 introduces relevant background knowledge including RFS, LRFS, and graph theory; Section 3 discusses how to determine the structure and formation of resolvable group targets in continuous and discrete environments. Then we carried out two simulations to verify our proposed method in Section 4. Finally, Section 5 is the summary of the paper.

## 2. Backgrounds

### 2.1. Labeled Random Finite Set (Labeled RFS)

In [30], Vo combines RFS with labels. RFS is essentially a set with random number of members, random state of members, and no fixed sorting rules among members. The random set is used to represent the target state of resolvable group targets. It can be expressed as Equation (1) at time *k*.

(1) $$X_{k}=\left\{x_{k,1},\cdots ,x_{k,N_{k}}\right\}.$$

In group target tracking, the situation of the target is very complex and changeable. For example, a certain target may suddenly disappear, or a new target may appear at a certain moment, or a target may be decomposed into multiple targets, and multiple targets may be combined into one target set. Therefore, Equation (1) can be expressed as Equation (2).

(2) $$X_{k}=\left[\underset{x\in X_{k-1}}{⋃}S_{k\left|k-1\right.}\left(x\right)\right]⋃\left[\underset{x\in X_{k-1}}{⋃}B_{k\left|k-1\right.}\left(x\right)\right]⋃\Gamma_{k},$$

where $S_{k\left|k-1\right.}\left(x\right)$, $B_{k\left|k-1\right.}\left(x\right)$, $\Gamma_{k}$ stand for the surviving targets, the spawned targets, and the birth targets, respectively. Similar to the state set, the measurement set of group targets is shown in the Equation (3).

(3) $$Z_{k}=\left\{z_{k,1},\cdots ,z_{k,M_{k}}\right\}.$$

RFS improved to labeled RFS is the addition of a unique tag $\ell \in L=\{\alpha_{i}:i\in N\}$ for each target $x_{k,i}$. N is the set of positive integers. We use the constraint function Equation (4) to guarantee the uniqueness of the label.

(4) $$\Delta \left(X\right)=\left\{\begin{array}{c} 1,\left|L\left(X\right)\right|=\left|X\right|\\ 0,\left|L\left(X\right)\right|\neq \left|X\right| \end{array}\right.$$

Reference [30] not only proposes the labeled RFS but also gives the densities of an LMB RFS and a labeled Poisson RFS. The LMB RFS’s density is described as:

(5) $$\begin{array}{c} \pi \left(\left\{\left(x_{1},l_{1}\right),\cdots ,\left(x_{n},l_{n}\right)\right\}\right)=\delta_{n}\left(\left|\left\{l_{1},\cdots ,l_{n}\right\}\right|\right)\times \prod_{\zeta \in \Psi }\left(1-r^{\zeta }\right)\prod_{j=1}^{n}\frac{1_{\alpha (\Psi )}\left(l_{j}\right)r^{\left(\alpha^{-1}\left(l_{j}\right)\right)}\left(x_{j}\right)}{1+r^{\left(\alpha^{-1}\left(l_{j}\right)\right)}} \end{array}.$$

### 2.2. Graph Theory

Graph theory has a wide range of applications. In this paper, we use directed graph to describe the structure of group. We take independent targets as vertices *V* of the graph *G* and relationships between targets as edges *E*. At the same time, we are describing this graph as a asymmetric adjacency matrix, which is the structure of the resolvable group target. The matrix is shown by the Equation (6).

(6) $$A_{d}=\left[\begin{array}{cccc} 0 & a(1,2) & \cdots & a(1,n)\\ a(2,1) & 0 & \cdots & a(2,n)\\ \vdots & \vdots & \ddots & \vdots \\ a(n,1) & a(n,2) & \cdots & 0 \end{array}\right],$$

Let target *i* be the parent node of target *j*, a(i,j)=1. If target *i* is target *j*’s child node, or target *i* has no relationship with target *j*, a(i,j)=0.

### 2.3. Graph Theory Model of Labeled RFS

For the relationship between any two vertex $v_{i}$, we can express it in terms of Equation (7).

(7) $$e_{i,j}:\left(x_{i},x_{j}\right)\to \left\{1,0\right\}.$$

Since each vertex has an unique label, we can simplify Equation (7) to Equation (8).

(8) $$e_{i,j}:\left(l_{i},l_{j}\right)\to \left\{1,0\right\}.$$

Equation (8) shows that the structure of the group is encapsulated by the graph defined on the target labels.

### 2.4. Revolvable Group Tracking with Maneuver and the Efficient Implementation for the GLMB Filter

Let target state be $x_{k,i}$ given as follows:

(9) $$x_{k,i}=\left[p_{k,x}\left(i\right),{\dot{p}}_{k,x}\left(i\right),p_{k,y}\left(i\right),{\dot{p}}_{k,y}\left(i\right)\right]\in X_{k}.$$

where $p_{k,x}\left(i\right)$ and ${\dot{p}}_{k,x}\left(i\right)$ are the position and velocity of target *i* on the *x*-axis, $p_{k,y}\left(i\right)$ and ${\dot{p}}_{k,y}\left(i\right)$ indicate the position and velocity of target *i* on the *y*-axis.

Suppose a target has a single parent node. We introduce the displacement $b_{k}\left(l,i\right)$ vector to describe this relationship and the resolvable group targets dynamic model is given as follows:

(10) $$\begin{array}{ccc} x_{k+1,i} & = & F_{k,l}x_{k,l}+b_{k}\left(l,i\right)+\Gamma_{k,i}\omega_{k,i} \end{array}$$

where $b_{k}\left(l,i\right)$, *l* contains the direction and distance information between the parent node *l* and child nodes *i*.

Therefore, for group targets, the displacement vector $b_{k}\left(l,i\right)$ can be represented as:

(11) $$\begin{array}{c} b_{k}\left(l,i\right)={\left[R_{k}\left(l,i\right)\times \cos\left(\theta_{k}\left(l,i\right)-\beta_{k}\left(l,i\right)\right),0,R_{k}\left(l,i\right)\times \sin\left(\theta_{k}\left(l,i\right)-\beta_{k}\left(l,i\right)\right),0\right]}^{T} \end{array},$$

where $R_{k}\left(l,i\right)$ denotes the designed distance between parent node *l* and child node *i*. $\beta_{k}\left(l,i\right)$ is the designed angle between nodes *l* and *i*. $\theta_{k}\left(l,i\right)$ is the motion direction for the parent target.

Under a maneuvering motion model, we assume that the formation of the group is stable within a certain time interval, so Equation (11) can be transferred to Equation (14).

(12) $$\begin{array}{c} b_{k}\left(l,i\right)=c_{k}\left(l,i\right)C_{a}\left(l,i\right)x_{k,l} \end{array}$$

(13) $$\begin{array}{c} c_{k}\left(l,i\right)=\frac{R(l,i)}{\sqrt{{\dot{p}}_{k,x}^{2}\left(l\right)+{\dot{p}}_{k,y}^{2}\left(l\right)}} \end{array}$$

(14) $$\begin{array}{c} C_{a}\left(l,i\right)=\left[\begin{array}{cccc} 0 & a_{\beta ,2}\left(l,i\right) & 0 & a_{\beta ,1}\left(l,i\right)\\ 0 & 0 & 0 & 0\\ 0 & a_{\beta ,1}\left(l,i\right) & 0 & -a_{\beta ,2}\left(l,i\right)\\ 0 & 0 & 0 & 0 \end{array}\right]. \end{array}$$

The derivation process has been described in detail in Reference [33].

Another point is the relation between covariance and adjacency matrix. To calculate the means and covariance, the parent vertex *l* should be first known. This is dependent on adjacency matrix Equation (6). In this paper, the adjacency matrix is defined on the label space and known in prior. That is, in the predicted stage, the adjacency matrix can be gotten and adopted according to the predicted labels. In contrast, if the adacency matrix is unknown, it needs to be estimated according to the predicted states. In general, the adacency relation is based on the target states, or the motion information. A detailed discussion can be found in Reference [36].

In the original GLMB filter [30,31], both the prediction step and the update step have their own independent truncation. This makes the computational complexity of GLMB filter very high. In order to optimize this problem, Vo et al. proposed the Gibbs GLMB in Reference [32]. The Gibbs GLMB algorithm combines the prediction and update steps of the GLMB filter so that there is only one truncation in each iteration. The density of the joint step of prediction and update step at time k is shown as:

(15) $$\pi_{k}\left(X\right)\propto \Delta \left(X\right)\sum_{J,\xi ,\theta }\left(\sum_{I}\omega_{k-1}^{\left(I,\xi \right)}\omega_{k}^{\left(I,\xi ,J,\theta \right)}\left(Z_{k}\right)\right)\delta_{J}\left(L\left(X\right)\right){\left[{p_{k}}^{\left(\xi ,\theta \right)}\left(\cdot |Z_{k}\right)\right]}^{X},$$

where

$$\omega_{k}^{\left(I,\xi ,J,\theta \right)}\left(Z_{k}\right)=1_{\Theta_{k}\left(J\right)}\left(\theta \right){\left[1-r_{B,k}\right]}^{B_{k}-J}{\left[r_{B,k}\right]}^{B_{k}\cap J}{\left[1-{\bar{P}}_{S,k}^{\left(\xi \right)}\right]}^{I-J}{\left[{\bar{P}}_{S,k}^{\left(\xi \right)}\right]}^{I\cap J}{\left[{\bar{\Psi }}_{Z_{k},k}^{\left(\xi ,\theta \right)}\right]}^{J}$$

$${\bar{\Psi }}_{Z_{k},k}^{\left(\xi ,\theta \right)}\left(l\right)=\langle \Psi_{Z_{k},k}^{\left(\xi ,\theta (l)\right)}\left(\cdot ,l\right),p_{k|k-1}^{\left(\xi \right)}\left(\cdot ,l\right)\rangle$$

$${\bar{P}}_{S,k}^{\left(\xi \right)}\left(l\right)=\langle P_{S,k}\left(\cdot ,l\right),p_{k-1}^{\left(\xi \right)}\left(\cdot ,l\right)\rangle$$

$${p_{k}}^{\left(\xi ,\theta \right)}\left(x,\cdot |Z_{k}\right)\propto \Psi_{Z_{k},k}^{\left(\xi ,\theta (l)\right)}\left(x,l\right)p_{k|k-1}^{\left(\xi \right)}\left(x,l\right)$$

$$p_{k|k-1}^{\left(\xi \right)}\left(x,l\right)=1_{B_{k}}\left(l\right)p_{(}B,k)\left(x,l\right)+1_{L_{k-1}}\left(l\right)\frac{\langle P_{S,k}\left(\cdot ,l\right)f_{k|k-1}\left(x|\cdot ,l\right),p_{k-1}^{\left(\xi \right)}\left(\cdot ,l\right)\rangle }{{\bar{P}}_{S,k}^{\left(\xi \right)}\left(l\right)},$$

Truncation by sampling ${\left\{\left(I^{\left(i\right)},\xi^{\left(i\right)},J^{\left(i\right)},\theta^{\left(i\right)}\right)\right\}}_{i=1}^{H_{k,max}}$ from some distribution π. It should be noted in the predicted density $p_{k|k-1}^{\left(\xi \right)}\left(\cdot ,l\right)$ that the collaboration noise $\omega_{k,i}^{o}$ is adopted, instead of process noise $\omega_{k,i}$.

## 3. Analysis of the Structure and Formation of Discernible Group Targets

Structures and formations exist in every aspect of our lives. For example, every bridge needs a suitable structure to carry its weight. The motorcade of the bride and groom is organized into a beautiful pattern, that is, a formation. In the military field, structures and formations are easier to spot. The design of structure and formation is a very important part of the air force, ground fighting vehicle force, and sea ship force, which affects the combat effectiveness of troops. This section describes in detail how to identify structures and formations of resolvable group targets.

### 3.1. The Structure and Formation of Resolvable Group Targets

#### 3.1.1. Structure

**Figure 1:**
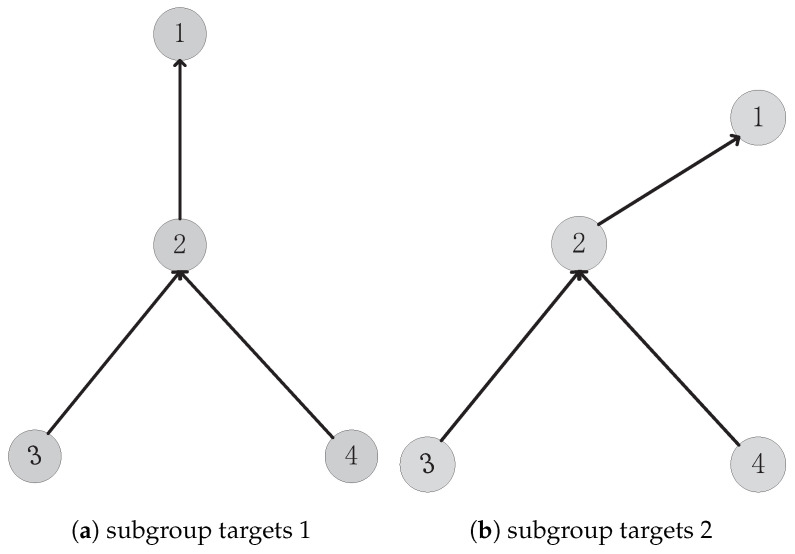
Two group targets with the same structure.

In this paper, the structure describes the collaborative relationship between the targets, we regard the collaboration between parent and child targets as the framework of the structure, while parent and child targets can be regarded as the important nodes of the structure. There is a detailed introduction on how to extract the structure of group targets in the Reference [35]. This paper focuses on how to determine whether the structure and formation of group targets have changed. First of all, let us talk about group structure in detail, focusing on when we think the group structure has not changed and when the group structure has changed. Since structure is the embodiment of collaboration, we can think of it this way: as long as the collaboration between group targets remains unchanged, the structure will remain unchanged. For example, in Figure 1, there are two independent subgroup targets, and each subgroup target has four subtargets. In these two subgroup targets, although the distance between its subtargets and the included angle of its relationship are different, their cooperative relationship is the same, so their structure is the same.

#### 3.1.2. Formation

The formation and structure of group objects are very similar. First of all, formation and structure are dependent on the collaborative relationship between sub-targets. If the cooperative relationship changes, formation and structure will inevitably change. The difference between the two is that formation has more strict standards than structure. For example, in a military parade in the queue, keep formation is in the process of the whole travel distance between the two fighters to almost keep constant, each consisting of three soldiers geometry angles that are almost the same. In the group of target formation, too, so we can regard it as a group of target formation and have a child between target space distance between the fixed and the geometry of fixed structure. Still here in Figure 1 as an example, two group of target Figure 1a and Figure 1b the same known structure, but target $x_{1}$ and $x_{2}$ in the Figure 1a the space distance and Figure 1b in the target space distance differences between $x_{1}$, $x_{2}$, and Figure 1a in the target consisting of $x_{1}$, $x_{2}$, $x_{4}$ geometrical angle and Figure 1b the angle of the corresponding target in also have bigger difference, so although the structure of the two group of consistent, but its formation is not consistent. In the process of judging, as long as there is a big difference between spatial distance and geometric angle, we can determine that the formation of the two groups is not consistent.

### 3.2. The Determination of Distinguishable Group Target Structure and Formation

In this paper, it is assumed that the relationship between father and son nodes in the group target will not be reversed, that is, the parent node will always be the parent node, will die, will separate, but will not become the child of its original child node.

#### 3.2.1. Determination of Group Target Structure in Continuous State

**Figure 2:**
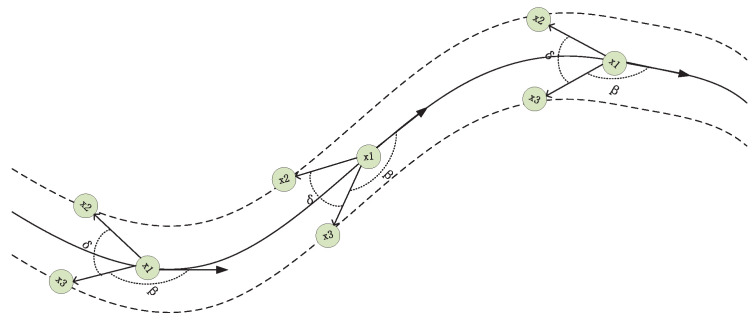
The trajectories of group targets under continuous conditions.

In this section we introduce the determination of group target structure in a continuous state. Based on the above description, we can clearly determine whether the elements of the structure are collaboration or not. The key to determine whether there is a collaboration relationship is the motion state of each subtarget and the spatial position between them. If the structure of a group is to remain in state, then the motion state of its members should be similar. If the motion state of a member differs greatly from that of other members, it is obvious that it must be separated from the group, and the structure of the group will also change accordingly. According to this principle, we can carry out a comparative analysis of its elements one by one. Firstly, in the discernible group target, the speed keeping approximation of all members is one of the important factors to judge whether the parent–child target cooperation relationship in the group target changes. We took the group target in Figure 2 as an example to demonstrate. At time *k*, the state of each target is $x_{k,i}$, and the velocity is $v_{k,i}$, then the state of target $x_{1}$, $x_{2}$, and $x_{3}$ at time k+1 can be expressed as:

(16) $$\begin{array}{c} x_{k+1,1}=x_{k,1}+v_{k,1}\\ x_{k+1,2}=x_{k,2}+v_{k,2}\\ x_{k+1,3}=x_{k,3}+v_{k,3} \end{array}$$

Therefore, the position relationship $L_{k}\left(i,j\right)$ between $x_{1}$ and $x_{2}$, $x_{1}$ and $x_{3}$, and $x_{2}$ and $x_{3}$ at time *k* and k+1 can be expressed as:

(17) $$\begin{array}{c} L_{k}\left(1,2\right)=x_{k,2}-x_{k,1}\\ L_{k}\left(1,3\right)=x_{k,3}-x_{k,1}\\ L_{k}\left(2,3\right)=x_{k,3}-x_{k,2} \end{array}$$

(18) $$\begin{array}{c} L_{k+1}\left(1,2\right)=x_{k+1,2}-x_{k+1,1}\\ L_{k+1}\left(1,3\right)=x_{k+1,3}-x_{k+1,1}\\ L_{k+1}\left(2,3\right)=x_{k+1,3}-x_{k+1,2} \end{array}$$

Substitute Equation (16) into Equation (18):

(19) $$\begin{array}{c} L_{k+1}\left(1,2\right)=x_{k,2}-x_{k,1}+\left(v_{k,2}-v_{k,1}\right)\\ L_{k+1}\left(1,3\right)=x_{k,3}-x_{k,1}+\left(v_{k,3}-v_{k,1}\right)\\ L_{k+1}\left(2,3\right)=x_{k,3}-x_{k,2}+\left(v_{k,3}-v_{k,2}\right) \end{array}$$

If the formation of the group is guaranteed to remain unchanged, the following conditions must be satisfied:

(20) $$L_{k+1}\left(i,j\right)=L_{k}\left(i,j\right)$$

That is:

(21) $$\begin{array}{c} v_{k,2}-v_{k,1}=0\\ v_{k,3}-v_{k,1}=0\\ v_{k,3}-v_{k,2}=0 \end{array}$$

Therefore, we can obtain the following conditions for the formation to remain stable:

(22) $$v_{k,1}=v_{k,2}=v_{k,3}$$

Therefore, we can draw a conclusion that, in the continuous state, the resolvable group targets can maintain stable structure and formation by judging whether the velocity of each subtarget in the group is consistent

The degree of approximation, we can use the speed $\delta_{vel}$ and speed-direction difference threshold $\delta_{dir}$ to limit speed. The deviation of velocity has cumulative, namely with the accumulation of time, while its speed is still approximate, but its space relative position deviation of the group members will be accumulated, so make sure that the collaboration will remain with the original, with its father–child node distance also needing to be tested, the distance threshold $\delta_{dis}$ is as shown in Equation (23):

(23) $$∥\left[\begin{array}{c} p_{x}^{1}\\ p_{y}^{1} \end{array}\right]-\left[\begin{array}{c} p_{x}^{2}\\ p_{y}^{2} \end{array}\right]∥≺\delta_{dis}$$

The constraint of the difference of velocity size is:

(24) $$∥\left[\begin{array}{c} {\dot{p}}_{x}^{1}\\ {\dot{p}}_{y}^{1} \end{array}\right]-\left[\begin{array}{c} {\dot{p}}_{x}^{2}\\ {\dot{p}}_{y}^{2} \end{array}\right]∥≺\delta_{vel}$$

The difference of velocity direction $\beta_{vdif}$ deviation is:

(25) $$\beta_{vdif}=\frac{{\dot{p}}_{y}^{1}}{{\dot{p}}_{x}^{1}}-\frac{{\dot{p}}_{y}^{2}}{{\dot{p}}_{x}^{2}}<\delta_{dir}$$

**Figure 3:**
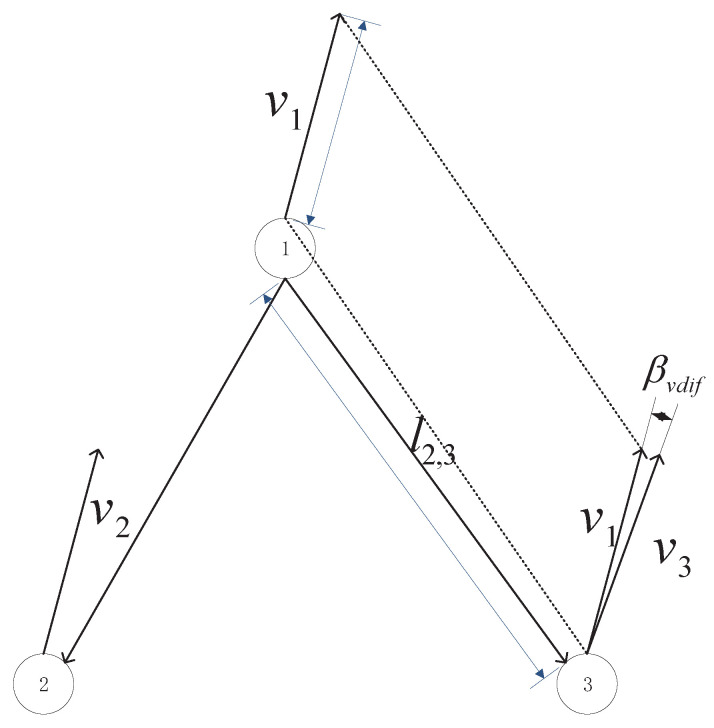
Schematic diagram of velocity in group target *v*, distance between father and son target $l_{ij}$, and velocity deviation $\beta_{vdif}$.

Through the above three constraints, we can determine the structure state of the group target, and the three elements of the structure are shown in Figure 3.

### 3.3. Determination of Group Target Formation in a Continuous State

**Figure 4:**
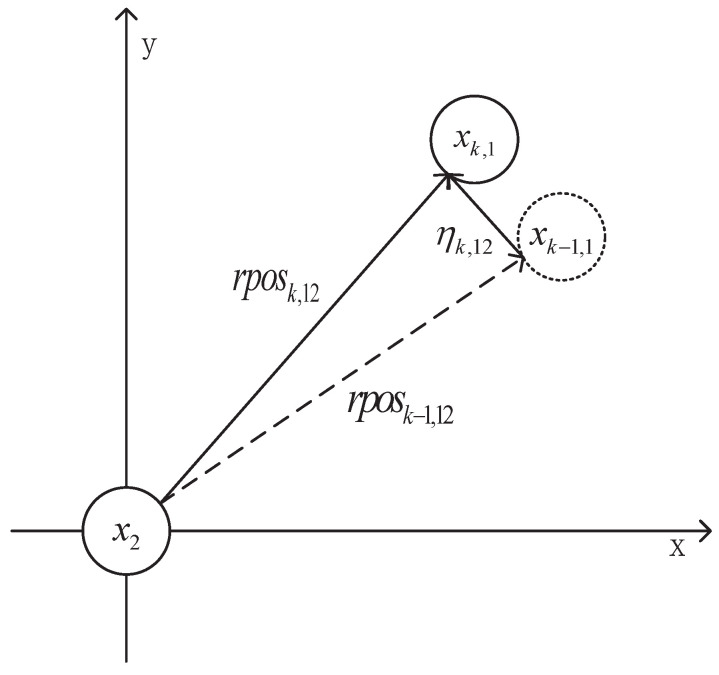
The schematic diagram of position deviation vector $\eta_{k,12}$.

The formation of the group target is a special case of the structure of the group target. Therefore, when analyzing the formation, we assume that the structure of the group target is unchanged. In other words, the speed factor of the group target member does not need to be considered, and we only need to judge whether the formation changes through its position state. In essence, it is to determine whether the relative position of two objects in a cooperative relationship has changed in two consecutive moments. The first step is to obtain the position deviation matrix $A_{P}$ of all members of the group target. the acquisition method is shown in the Equation (26).

(26) $$A_{p}={\left[\begin{array}{cccc} 0 & ∥\eta_{12}∥ & \cdots & ∥\eta_{1n}∥\\ ∥\eta_{21}∥ & 0 & \cdots & ∥\eta_{2n}∥\\ \vdots & \vdots & \ddots & \vdots \\ ∥\eta_{n1}∥ & ∥\eta_{n2}∥ & \cdots & 0 \end{array}\right]}_{n\times n}$$

where *n* is the number of group members, and $\eta_{ij}$ is the deviation vectors of the relative position vectors of the two targets in the group at time *k* and time k−1. It can be seen from Figure 4 that the position deviation vector $\eta_{ij}$ can be obtained from the displacement vector rpos of the target and the target at time *k* and time k−1. The calculation method is as follows:

(27) $$\eta_{k,ij}={rpos}_{k,ij}-rpos_{k-1,ij}$$

(28) $$rpos_{k,ij}=\left[\begin{array}{cccc} 1 & 0 & 1 & 0 \end{array}\right]*\left(x_{k,i}-x_{k,j}\right)$$

After obtaining position deviation matrix $A_{P}$, we can obtain the formation change coefficient of group target through Equation (29).

(29) $$\varphi =∥A_{k}A_{P}{∥}_{2}$$

$A_{k}$ is the adjacency matrix describing the group structure. Through $A_{k}$, the data with collaboration relationship targets in the group are screened out from $A_{p}$. According to the observation, we set a threshold of formation coefficient $\sigma_{dis}$. If the coefficient Φ is greater than the threshold $\sigma_{dis}$, the formation of group targets is considered to be fixed; if not, it is considered to have not changed, and the relationship is shown in the formula.

(30) $$\left\{\begin{array}{cc} 1, & \Phi >\sigma_{dis}\\ 0, & \Phi \leq \sigma_{dis} \end{array}\right.$$

## 4. Simulations

### 4.1. Experiment 1

#### 4.1.1. Configuration Parameters for Experiment 1

**Figure 5:**
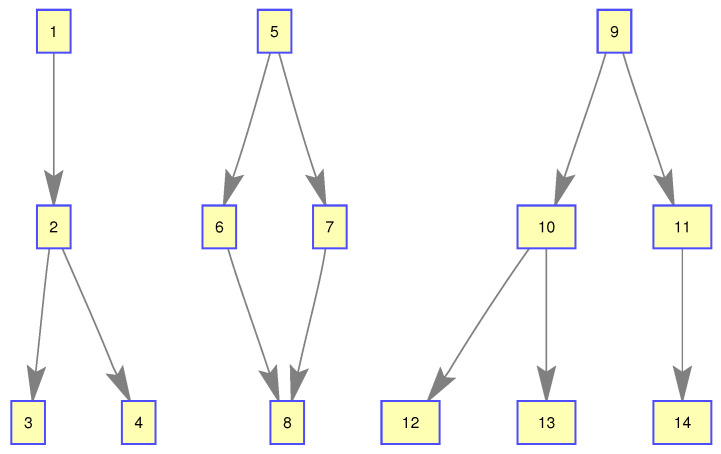
The structure of subgroup targets.

In this experiment, we used a Gibbs-GLMB filter to get the state estimation of group targets. There are three subgroups of group target, including 4, 4, and 6 members, respectively. Their structure is shown in Figure 5.

The initialized state of the distance between any parent and child targets is 100 meters. $\left\{\left(x_{1},\ell_{1}\right),\cdots ,\left(x_{4},\ell_{4}\right)\right\}$ is subgroup 1, $\left\{\left(x_{5},\ell_{5}\right),\cdots ,\left(x_{8},\ell_{8}\right)\right\}$ is subgroup 2, and $\left\{\left(x_{9},\ell_{9}\right),\cdots ,\left(x_{14},\ell_{14}\right)\right\}$ is subgroub 3. The three subgroups are independent of each other.In the initial state, the adjacency matrix of the three subgroups is set as known in this paper, which can be expressed as:

(31) $$A_{1}\left(\ell_{1},\cdots ,\ell_{4}\right)=\left[\begin{array}{cccc} 0 & 0 & 0 & 0\\ 1 & 0 & 0 & 0\\ 0 & 1 & 0 & 0\\ 0 & 1 & 0 & 0 \end{array}\right]$$

(32) $$A_{2}\left(\ell_{5},\cdots ,\ell_{8}\right)=\left[\begin{array}{cccc} 0 & 0 & 0 & 0\\ 1 & 0 & 0 & 0\\ 1 & 0 & 0 & 0\\ 0 & 1 & 1 & 0 \end{array}\right]$$

(33) $$A_{2}\left(\ell_{9},\cdots ,\ell_{14}\right)=\left[\begin{array}{cc} \begin{array}{ccc} 0 & 0 & 0\\ 1 & 0 & 0\\ 1 & 0 & 0 \end{array} & \begin{array}{ccc} 0 & 0 & 0\\ 0 & 0 & 0\\ 0 & 0 & 0 \end{array}\\ \begin{array}{ccc} 0 & 1 & 0\\ 0 & 1 & 0\\ 0 & 0 & 1 \end{array} & \begin{array}{ccc} 0 & 0 & 0\\ 0 & 0 & 0\\ 0 & 0 & 0 \end{array} \end{array}\right]$$

**Figure 6:**
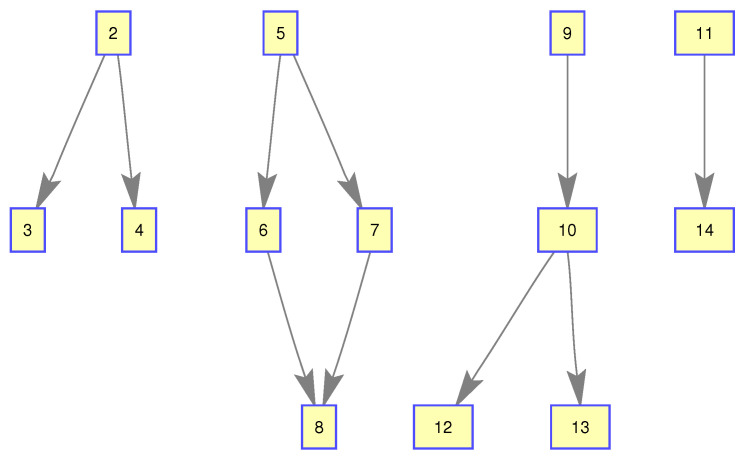
The structure of subgroup targets during k=30 and k=70.

**Figure 7:**
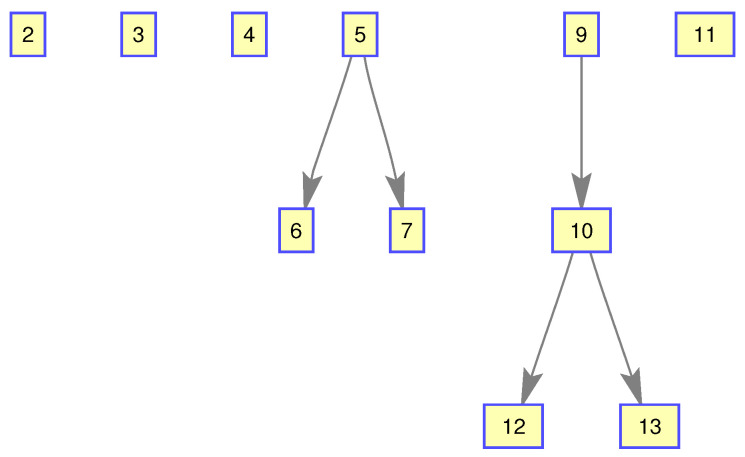
The structure of subgroup targets during k>70.

**Figure 8:**
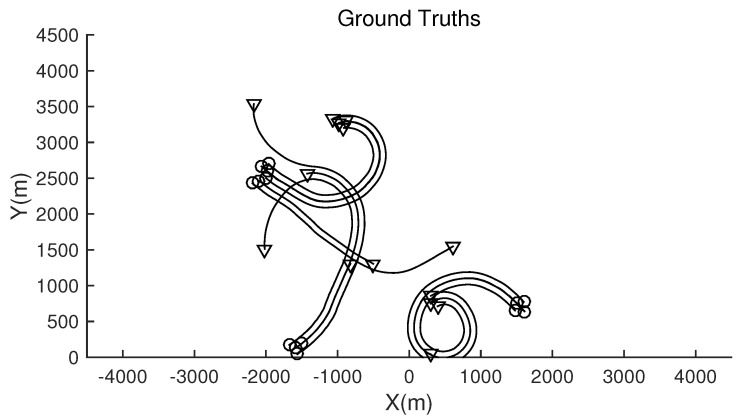
The structure of subgroup targets during k>70.

The monitoring scope of experiment 1 is [−π/2,π/2;0m,3000m]. The duration of the experiment was 100 s: subgroup 1 was born at k=0 s, subgroup 2 and subgroup 3 were both born at k=20 s, target $x_{1}$, the head node in subgroup 1, died at k=30 s, target $x_{8}$ in subgroup 2 and target $x_{11}$ in subgroup 3 died at k=70 s. In terms of structure, subgroup 3 is decomposed into two subgroup targets at k=30 s. Target $x_{11}$ and its child target $x_{14}$ are separated from subgroup 3 into subgroup 4. At this time, subgroub 4 only has target $x_{11}$ and target $x_{14}$ and the dependency between target $x_{11}$ and target $x_{14}$ is the same as before. At k=70 s, target $x_{2}$, $x_{3}$, and $x_{4}$ of subgroup 1 are completely separated into three independent targets, and target $x_{11}$ of subgroup 4 becomes an independent target. The structure between k=30 and k=70 s is shown in Figure 6, and the structure of the group at k>70 s is shown in Figure 7. The covariance of the observed noise is $R=\mathrm{diag}[\begin{array}{cc} 0.0012 & 100 \end{array}]$. The covariance of process noise is $Q=\mathrm{diag}[\begin{array}{ccc} 0.04 & 0.04 & 0.04 \end{array}]$. The real trajectory of the target is shown in Figure 8. The curve represents the trajectory, the circle represents the starting point, and the triangle represents the ending point.

#### 4.1.2. The Result of the Experiment 1

**Figure 9:**
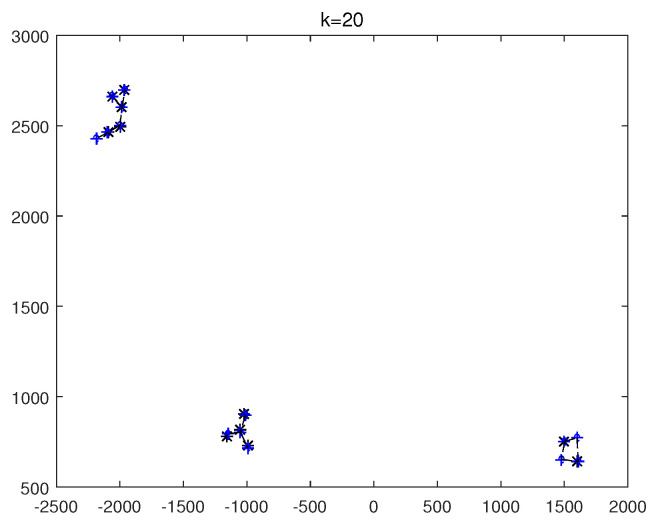
The state estimation of group targets at k=20.

**Figure 10:**
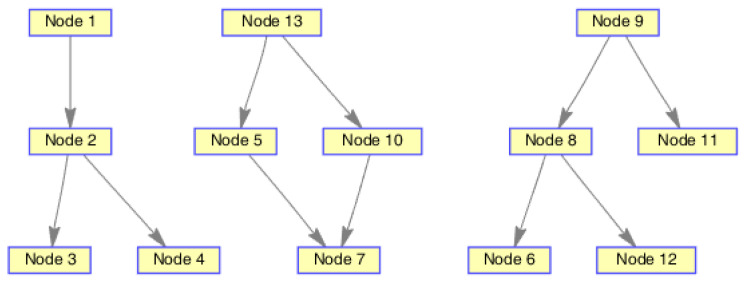
The structure estimation of group targets at k=20.

**Figure 11:**
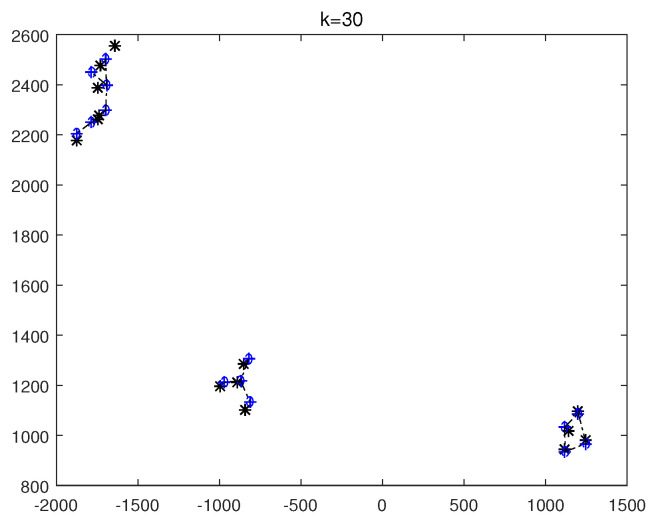
The state estimation of group targets at k=30.

**Figure 12:**
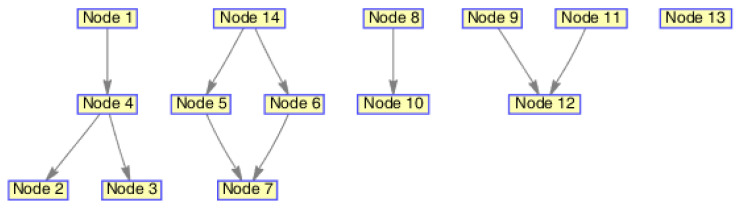
The structure estimation of group targets at k=30.

**Figure 13:**
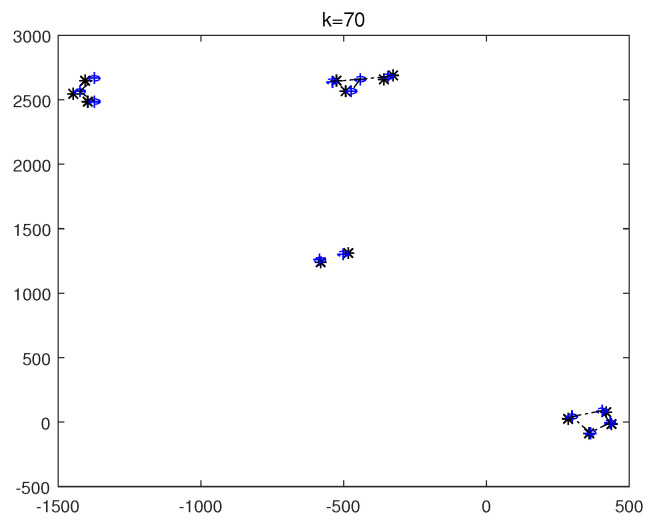
The state estimation of group targets at k=70.

**Figure 14:**
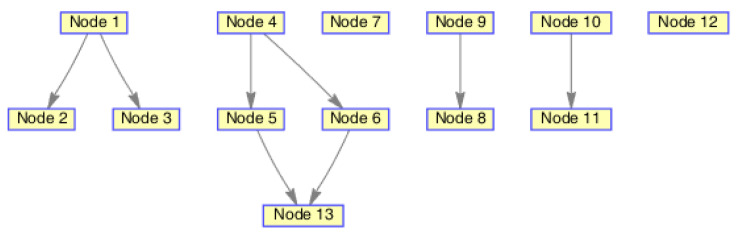
The structure estimation of group targets at k=70.

In this experiment, we set that when the structure of the group target is detected to change, the estimated point of the group target and the structure of the group target at this time are immediately output. In the experimental environment parameter configuration, we can get that the group structure will change significantly when k=20, k=30, and k=70. At k=20, 10 targets will be born. At k=30, a target dies, and subgroup 3 splits into two subgroups. At k=70, there is both the death of the target and the change in the target structure. Therefore, we focus on the detection results of these three moments from the results. The following are the experimental result figures of the three moments in this experiment. When k=20, the position of the target and the structure of the group target are shown in Figure 9 and Figure 10. In Figure 9, we can see that Gibbs-GLMB tracks 13 points at this point, compared to k=19, there are 9 target regenerations. Although the collaboration relationship between the targets has not changed compared with the previous moment, the overall structure of the group is bound to change with the increase of the members of the group, so the tracked target is traced and its structure is output. When k=30, the head node of a subgroup dies, and a subgroup is separated into two subgroups, and the change is also detected successfully, as shown in Figure 11 and Figure 12. When k=70, the detection results are shown in Figure 13 and Figure 14.

**Figure 15:**
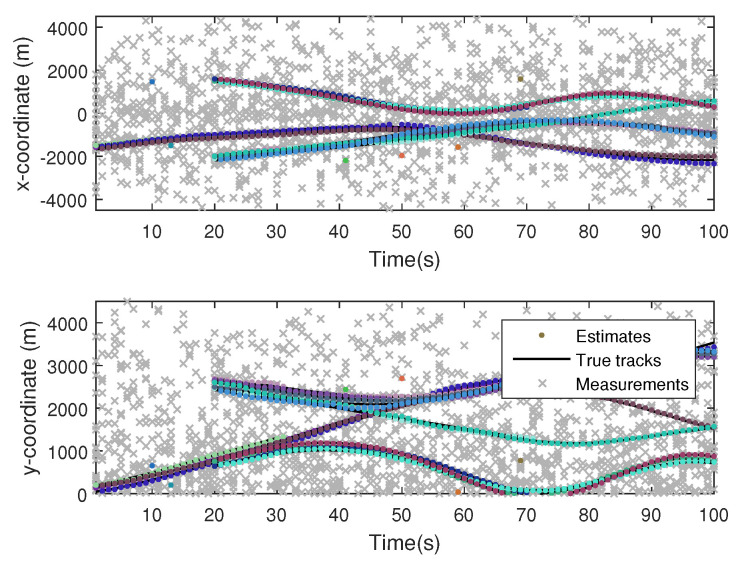
The state estimation by Gibbs Generalized Labeled Multi-Bernoulli (GLMB) filter.

**Figure 16:**
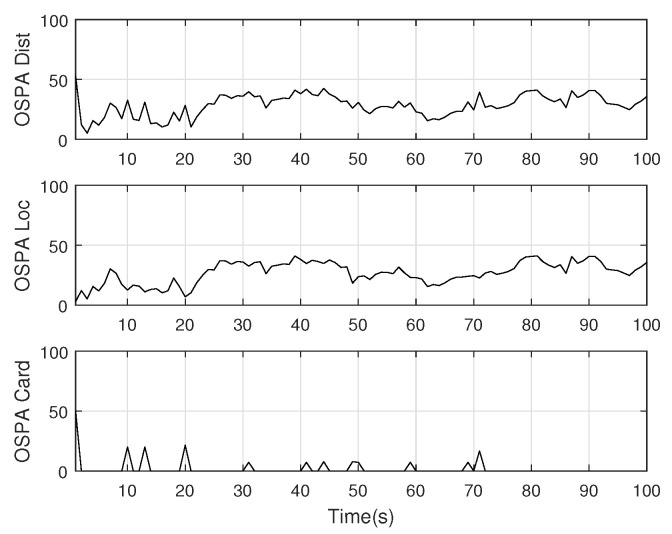
The OSPA distance by Gibbs-GLMB filter.

**Figure 17:**
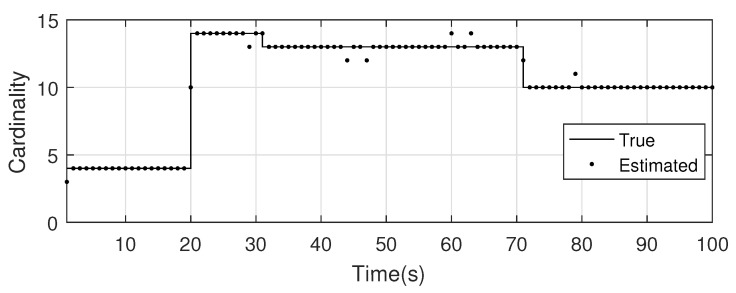
The estimated number of targets by GLMB filter.

The position state estimation of Gibbs-GLMB filter, OSPA distance, and target number estimation are shown in Figure 15, Figure 16 and Figure 17.

The results show that the proposed method can effectively identify the change of group target structure, but there are some problems, such as false alarm. As a result that the method relies on Gibbs GLMB state estimation results, when the state estimation error occurs, the structure estimation error will also occur. In addition, the threshold setting too large or too small will cause error, the rationality of the threshold setting is one of the problems worth in-depth study.

### 4.2. Experiment 2

#### 4.2.1. Configuration Parameters for Experiment 2

**Figure 18:**
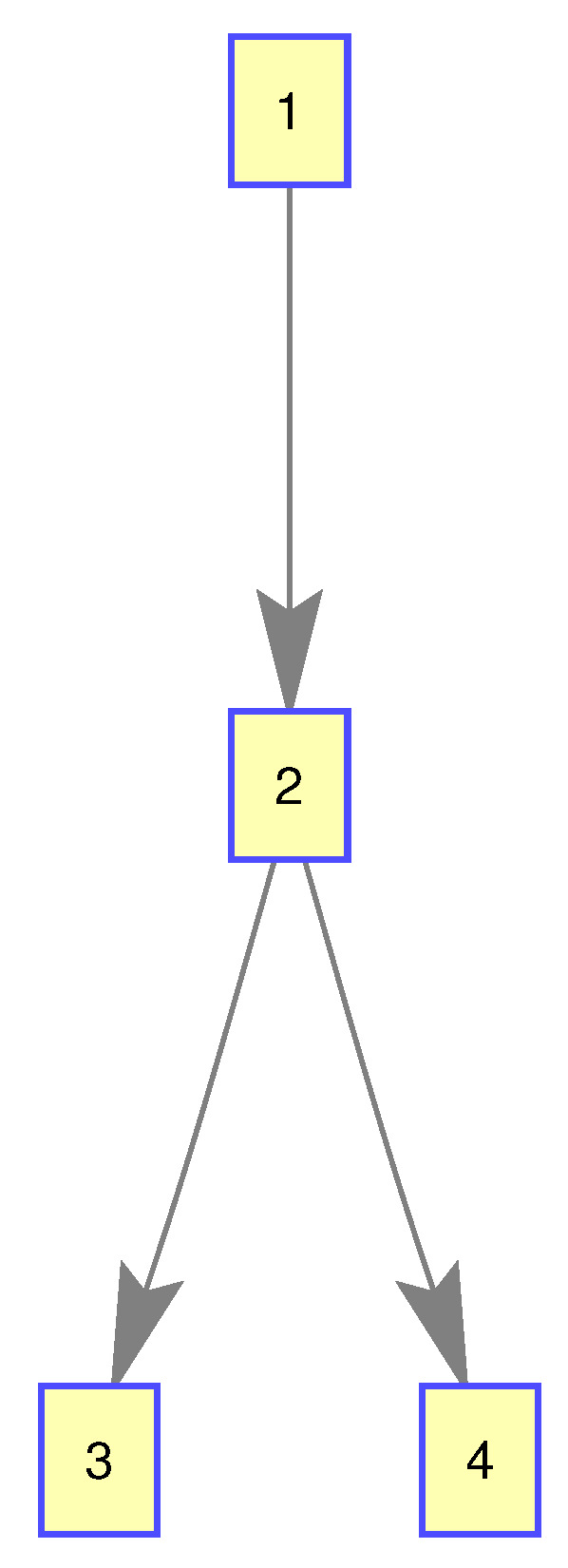
The structure of the single group targets.

In this experiment, we only selected a single group target with four members for the experiment, whose structure is shown in the Figure 18:

The distance between the parent and child targets set in the initial state is 100 m, the same as in experiment 1. The adjacency matrix of the group $\left\{\left(x_{1},\ell_{1}\right),\cdots ,\left(x_{4},\ell_{4}\right)\right\}$ is:

(34) $$A_{1}\left(\ell_{1},\cdots ,\ell_{4}\right)=\left[\begin{array}{cccc} 0 & 0 & 0 & 0\\ 1 & 0 & 0 & 0\\ 0 & 1 & 0 & 0\\ 0 & 1 & 0 & 0 \end{array}\right]$$

**Figure 19:**
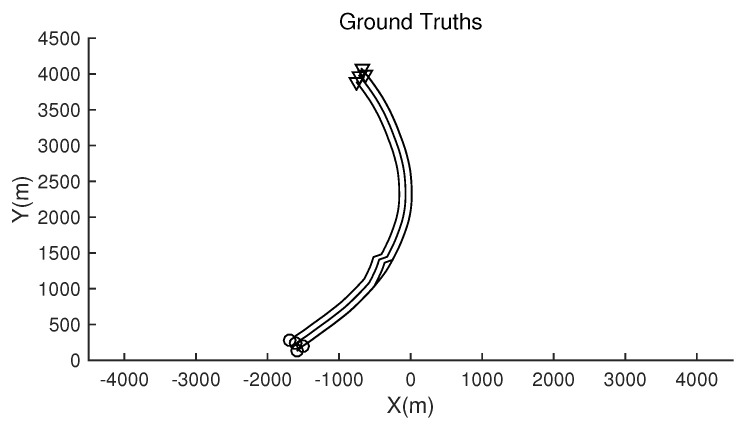
The track of the single group targets.

The other parameters are basically the same as those in experiment 1, except that there are no birth, death and ovulation of the target in this experiment. As a result that this experiment studies the change of formation, we assume that its structure is stable and unchanged, which can simplify our modeling. In the model, we increase the distance between target $x_{1}$ and target $x_{2}$ in the group by 10m per second between k=30 and k=40 s. In other words, the formation of the group will continue to change during this period, and its track is as Figure 19:

#### 4.2.2. The Result of the Experiment 2

**Figure 20:**
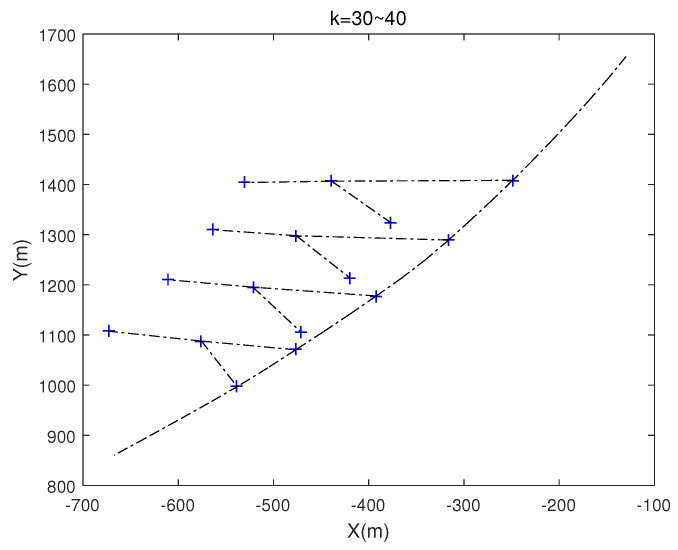
The position of the target at k=30 to k=40.

When a change in formation is detected, the current and previous positions of the group target are plotted immediately.The results show that all the preset change nodes can be detected successfully, and the detected position diagram at the time k=30 to k=41 is shown in Figure 20. The blue cross represents the current target position and the red circle represents the previous target position.

The experiment was carried out for ten times, and the detection accuracy of ten times was shown in Table 1. According to the data in the Table 1, we can know that the average recognition accuracy of the ten experiments is 74.70%.

## 5. Conclusions

In this paper, we analyze the structure and formation of group targets based on the information of target position and velocity, and then propose a method to determine whether they have state changes. First, we use the Gibbs-GLMB filter to estimate the state of group target. Then, by analyzing and comparing the state estimation data of the target, we can determine whether its structure or formation has changed. The structure problem is mainly determined by the distance between the targets, the velocity difference between the two targets, and the angle difference between the velocity direction and the position vector. For the formation problem, it is mainly determined by the distance between the targets and the offset angle difference of the position vector. Finally, experiments show that the method proposed in this paper can effectively identify the structure and formation changes of group targets.

## Figures and Tables

**Table 1:** The judgment precision of ten experiments and the average value of ten experiments.

No.	1	2	3	4	5	6	7	8	9	10
precision	78.57%	64.71%	84.62%	68.75%	64.71%	81.82%	73.33%	78.57%	73.33%	78.57%
average	74.70%									
